# Determination of Aluminum in Dialysis Concentrates by Atomic Absorption Spectrometry after Coprecipitation with Lanthanum Phosphate

**Published:** 2017

**Authors:** Emine Kılıçkaya Selvi, Uğur Şahin, Serkan Şahan

**Affiliations:** a *Recep Tayyip Erdoğan University, Faculty of Art and Science, Chemistry Department, 53100, Rize, Turkey.*; b *Erciyes University, Faculty of Science, Chemistry Department, 38039, Kayseri, Turkey. *; c *Erciyes University, Faculty of Agriculture, Department of Soil Science and Plant Nutrition , 38039, Kayseri, Turkey.*

**Keywords:** Aluminum, Lanthanium phosphate, Coprecipitation, Dialysis concentrate, Atomic absorption spectrometer

## Abstract

This method was developed for the determination of trace amounts of aluminum(III) in dialysis concentrates using atomic absorption spectrometry after coprecipitation with lanthanum phosphate. The analytical parameters that influenced the quantitative coprecipitation of analyte including amount of lanthanum, amount of phosfate, pH and duration time were optimized. The % recoveries of the analyte ion were in the range of 95-105 % with limit of detection (3s) of 0.5 µg l^-1^. Preconcentration factor was found as 1000 and Relative Standard Deviation (RSD) % value obtained from model solutions was 2.5% for 0.02 mg L^-1^. The accuracy of the method was evaluated with standard reference material (CWW-TMD Waste Water). The method was also applied to most concentrated acidic and basic dialysis concentrates with satisfactory results.

## Introduction

Aluminum is an essential metal element in biological systems. It exists in nature extensively and it is one of the most abundant elements on the earth’s crust. Before 1970 s, aluminum and aluminates were treated as substances which could not be absorbed, and were safe without any poisonous effects. 

Therefore, aluminum and aluminates were widely used in food additives medicines, water coagulants and all kinds of cookers and containers. From mid-70s, with the development of analytical techniques and the increasing reports about the poisonous effects of aluminum, the study about the poisonous effects of aluminum to human beings had been further developed. In fact, when people ingested high amounts of aluminum ions, aluminum would deposit in the human bodies, and interfered with the normal activities of nervous system (1) and cause brain diseases, like language difficulties, movement obstructions and abnormal electroencephalogram (2) Some regions of the brain associated with neurofibrillary degeneration in patients with Alzheimerʹs disease have been shown to have 10 to 30 time normal aluminum concentration (3). 

The literature records several analytical techniques for the determination of aluminum. Spectrophotometry (4), spectrofluorimetry (5-9), inductively coupled plasma optical emision spectrometry (ICPOES) (10-12), inductively coupled plasma mass spectrometry (ICPMS) (13, 14). electrothermal atomic absorption spectrometry (ETAAS), (15-19), chromatography (20,21) electroanalytical methods (22, 23), diffus reflectance spectroscopy (24,25), and flameatomic absorption spectrometry (FAAS) (26-28) are widely used for determination of aluminum in various samples. The dialysis fluids are prepared from dialysis concentrates that are mixed with pure water. If aluminium is present as a contaminant in these fluids, it is able to diffuse through the dialysis membranes and penetrate into the blood stream of the patient. 

**Figure 1:**
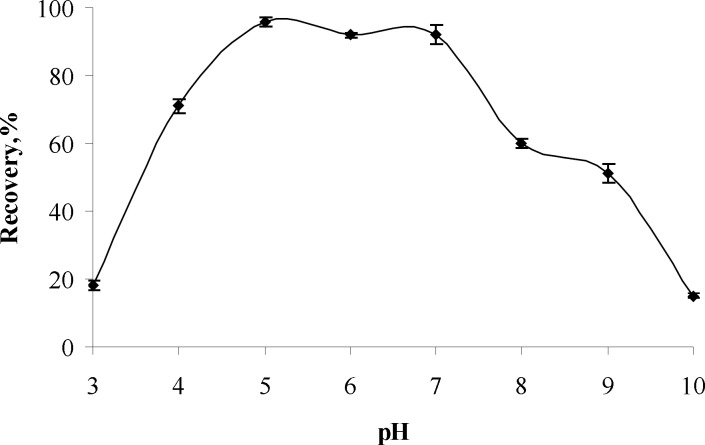
The pH effects on recoveries of aluminum(III) (n=3).

**Figure 2.**
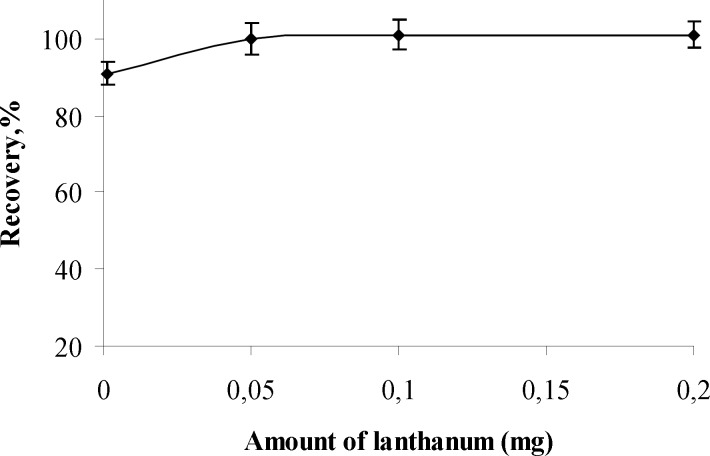
The effects of amount of lanthanum (III) on the recoveries of analyte ions (n=3

**Figure 3:**
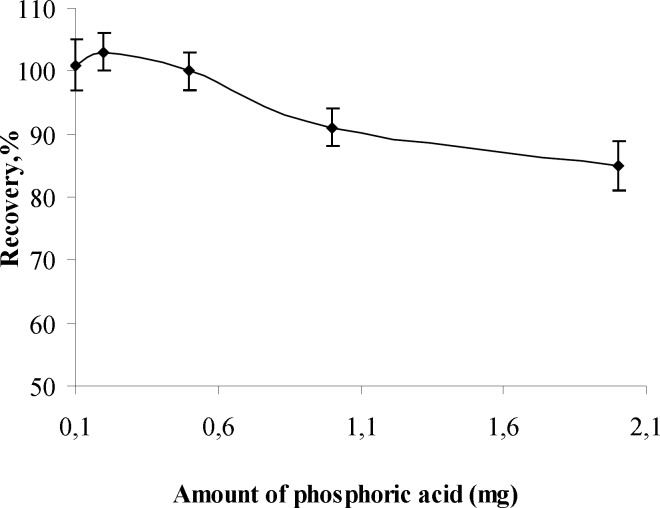
The effects of amount of phosphoric acid on the recoveries of analyte (n=3

**Figure 4:**
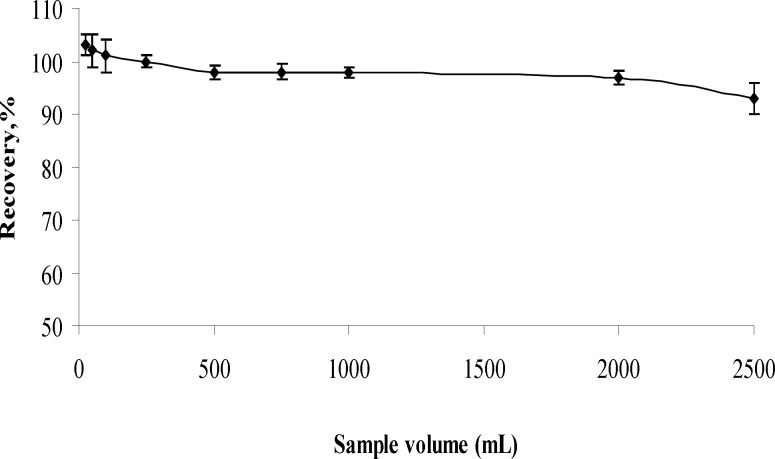
Effects of sample volume on recoveries of analyte (n=3

**Table 1:** The determination of aluminum in the standard reference material (n= 3).

**Sample**	**Certified value**	**Found**	**Recovery%**
CWW-TMD Waste water	1.00 ± 0.05	1.01 ± 0.03	101 ± 3

**Table 2:** Determination of aluminum (III) in spiked solutions (n=3).

	**Added (mg L** ^-1^ **)**	**Found (mg L** ^-1^ **)**	**Recovery%**
A1*	- 5.0	8.3 ± 0.1 13.2 ± 0.2	- 99 ± 2
A2	-	18.7 ± 0.2	-
A3	-	16.2 ± 0.2	-
B1**	- 5.0	1.7 ± 0.1 6.7 ± 0.2	- 100 ± 2
B2	-	1.5 ± 0.1	-

* Acidic dialysis concentrate

** Basic dialysis concentrate

The contamination levels in these cases depend strongly on the quality of the water and the dialysis concentrates used in the dialysis fluid preparation. The official pharmacopeias require an accurate control of the trace levels of aluminium in commercial dialysis solutions, which must be lower than 10 µg L^−1^ (29). 

The problem with the analysis of the concentrates is very high salt concentration of these solutions. Typically they contain about 400 gL^-1^ of sodium, potassium, calcium, magnesium chloride and sodium acetate. Therefore separation is necessary to analyse aluminum in dialysis concentrates accurately. Some of the major methods of separation and preconcentration involve evaporation,volatilization,coprecipitation, cloud point extraction, solvent extraction and solid-phase extraction (30-38).

La (III) was used as releasing agent and ion suppressor in flame for determination of metal ions (39, 40). LaPO_4 _was used as coprecipitant for separation and preconcentration of heavy metals in some water samples (41). According to our knowledge, LaPO_4_ was firstly used for separation and preconcentration of aluminum in dialysis concentrates with this study. This method has several advantages such as low detection limit (DL), simple, rapid, economic and precise. The recoveries of aluminium (III) in the presence of the most common matrix elements containing the alkaline and alkaline earth metals were good.

*Influence of amount of lanthanum (III) as carrier element*

The influence of amounts of lanthanum (III) as carrier element on recoveries of aluminum (III) ion was examined at pH 5 while keeping other parameters constant. 

The results are given in Figure.2 and shows that the quantitative recoveries of analyte ions were obtained at the lanthanum (III) amounts range of 0.05 – 0.2 mg. The optimum amount of lanthanum (III) was kept constant as 0.15 mg in further experiments.

## Result and Discussion

*Influence of pH*

The influence of pH on recoveries of analyte ion were investigated in the pH range of 3-10 and the result are given in Figure 1 It can be concluded from the Figure. that the highest recovery (95 %) of analyte ions were obtained at pH 5 and further experiments works were performed at pH 5.

*Influence of amount of phosphoric acid*

The influence of amount of phosphoric acid on the recovery of aluminum (III) ion was also investigated and the results are given shown in Figure.3. It can be concluded from the Figure 3 that the quantitative recoveries of Al (III) in the range of 0.1-2 mg of phosphoric acid (1:5 D). There fore, 0.5 mg of phosphoric acid was the optimum amount investigated and used in further experiments of the proposed method.

*Influence of standing time*

The influence of standing time for the coprecipitation on recoveries of analyte ion was studied in the time range of 5 – 60 min. While keeping all the other parameters constant, quantitative recoveries for analyte were obtain at 15 min of standing time. All the other experiments of the proposed method were performed at 15 min of standing time.

*Influences of centrifugation time and rate*

The effect of centrifugation rate on the recoveries of analyte was studied in the range of 1500 – 4000 rpm. While keeping centrifugation time constant, maximum recoveries of Al (III) was obtained at the rate of 3500 rpm. The influences of centrifugation time on recoveries of analyte ion were investigated in the range of 5 – 30 min at 3500 rpm. The quantitative recoveries for aluminium (III) ions were obtained with 10 min of centrifugation time. The further experiments were performed at 3500 rpm for 10 min.

*Influence of sample volume*

The recoveries of aluminum (III) from different sample volumes were tested in sample volume range of 25 – 2000 mL. The results are given in Figure 4. For 25-50 mL of sample volumes, the precipitates formed in polyethylene tube and solutions were separated each other with centrifugation. For above 50 mL of sample volumes, the precipitates were filtered throughout cellulose nitrate membrane. Analyte ion were quantitatively (95%) recovered in the all sample volume. Further experiments were performed by maintaining 500 mL volume of the final solution.

*Analytical figures or merit*

The characteristic data for the performance of the method under the optimum conditions were studied. Limit of detection (LOD) of the proposed procedure for the determination of analyte (n=21) was found to be as 0.5 µg.L^-1^ with 3s criterion. Preconcentration factor was found as 1000 (from 2000 mL to 2 mL). RSD % value obtained from model solutions was 2.5% for 0.02 mg L^-1^.

*Accuracy of the method*

The accuracy of the method was tested with CWW-TMD Waste Water certified reference material (CRM). 10 mL of CRM sample was pipetted to a beaker and dissolved to 100 mLapproximately. 

The pH value of this solution was adjusted to 5 with diluted HCl and NaOH solutions. This solution was analyzed using the preconcentration procedure describe above. The results obtained from CRM were given in Table 1

*Application of the method*

The proposed method was applied to the analysis of Al (III) in various acidic and basic dialysis solutions with satisfactory results. Addition recovery experiments for aluminum (III) were performed to two dialysis solution and the results are shown in Table 2. 

There is a good agreement between added and measured aluminum amounts which confirm that the method can be successfully applied to dialysis solutions. The recoveries for the additions of 5 mgl^−1^ of Al(III) were 99% to 100%.

## Experimental

*Instruments*

A Perkin-Elmer Analyst A800 Model atomic absorption spectrometer (Northwalk, USA) with nitrous oxide/acetylene flame and a D_2_ lamp with background corrector was used throughout the determination of aluminum (III) in model solutions and samples.

All the instrumental settings were run as recommended by manufacturer like (wavelenght was 309.3 nm, slit width was 0.7 nm and lamp current was 25 mA). A Consort C533 Model pH meter (Turnhout, Belgium) was used for measuring pH values in the aqueous phase. ALC PK 120 model centrifuge (Milan, Italy) was used for centrifugation of sample and model solutions. The water was purified by Elga (Bucks, UK) water purification system.

*Chemicals*

All chemicals used in this work were of analytical reagent grade. Laboratory glassware was kept for 1 day in a 10% HNO_3 _solution and then cleaned with deionized water.

Stock solutions of 1000 µg mL^-1^ in 1 mol l^-1^ HNO_3_ solution of Al(III) was used for the preparation of standard and model solutions. The acetate/acetic acid buffer solution.was used to obtain pH 5.Membrane with pore size of 0.45 µm was used for filtration purpose of all the sample solutions.

*General Procedure*

For coprecipitation 2 µg aluminum (III), 150 µg lanthanum (III) and 150 µL phosphoric acid ( 1:2 diluted water) was placed in a centrifuge tube. Then the pH of the solution was adjusted to pH 5 with ammonium acetate/ acetic acid and the solution was diluted to 50 mL with distilled water. After shaking the solution for several seconds the solution was allowed to stand for 15 min and centrifuged at 3500 rpm for 15 min. The supernatant was removed and the precipitate in the tube was dissolved with 0.1 mL of concentrated HNO_3_ and the volume was completed to 2 mL with distilled water. The number of replicates for each analysis was three and the analyte in the solution was determined with flame atomic absorption spectrometry.

*Analysis of dialysis concentrate*

The dialysis solutions collected from a local drug company in Kayseri, Turkey. The concentration (g L^-1^) of each component was as follows.

(1) Concentrated acidic solution :

NaCI 214.77; KCI 5.22; CaCI_2_.2H_2_O 7.72; MgCI_2_.6H_2_O 3.56; CH_3_COOH 4.20.

(2) Concentrated basic solution: NaHCO_ 3_ 84.0.

During the analysis acidic dialysis concentrate (500 mL) the pH was adjused to 5 with NaOH and HCI, the solution was analysed using the same preconcentration procedure describe earlier. The acidic dialysis solutions were filtered through cellulose membrane filter. The precipitate was dissolved with 1 mL of concentrated HNO_3_ and evaporated on hot plate until dry residue were obtained. The residue was dissolved with 1 mol L^-1^ HNO_3_ solution and diluted to 2.0 mL with the same solution.

During the analysis of basic dialysis concentrate (500 mL), at first time pH adjusted to 2 with HCI and solution was boiled on hot plate until CO_2 _was removed. Then solution was allowed to cool down and the pH was adjusted to 5 with NaOH and HCI. This solution was also analyzed using the preconcentration procedure describe above. The basic dialysis solution was filtered through a cellulose membrane filter and the precipitate was dissolved with 1 mL of concentrated HNO_3_ and evaporated on hot plate until dry residue were obtained. The residue was dissolved with 1 mol L^-1^ HNO_3_ solution and diluted to 2.0 mL with the same solution. The acidic and basic concentrates were spiked with known concentration of Al (III).

## Conclusion

The coprecipitation procedure with lanthanum phosphate examined in this work provides a very rapidly simple precise and reliable technique for the preconcentration of aluminum (III) from dialysis samples. Lanthanum was used for both coprecipitant and ion suppressor and used as releasing agent in flame. The quantitative recovery values were obtained from real samples and certified reference material. The coprecipitated analyte ion can be precisely determined by atomic absorption spectrometry without any influence of lanthanum phosphate. As there is no interferences effect of the content of dialysis concentrates solutions the influence of matrix effect was not investigated. 

As it is seen in the results, the concentration of aluminum (III) in acidic dialysis concentrate is higher than the basic dialysis concentrate. Although basic concentrate contains only sodium bicarbonate or in addition sodium chloride, acidic concentrate contains many chloride salts, dextrose and acetic acid. The same equipment and pure water are used in production of the acidic and basic dialysis concentrates. Therefore the main reason of rising of aluminum(III) level in the acidic concentrate is the raw materials used in the production.
